# The Role of RNA Binding Proteins for Local mRNA Translation: Implications in Neurological Disorders

**DOI:** 10.3389/fmolb.2019.00161

**Published:** 2020-01-15

**Authors:** Maximilian Paul Thelen, Min Jeong Kye

**Affiliations:** Institute of Human Genetics, University of Cologne, Cologne, Germany

**Keywords:** ribonucleoproteins, RNA binding proteins, mRNA translation, local protein synthesis, SMA, ALS, FXS, neurodegeneration

## Abstract

As neurons are one of the most highly polarized cells in our body, they require sophisticated cellular mechanisms to maintain protein homeostasis in their subcellular compartments such as axons and dendrites. When neuronal protein homeostasis is disturbed due to genetic mutations or deletions, this often results in degeneration of neurons leading to devastating outcome such as spinal muscular atrophy (SMA), amyotrophic lateral sclerosis (ALS), and fragile X syndrome (FXS). Ribonucleoprotein (RNP) complexes are macromolecular complexes composed of RNA binding proteins (RBPs) and their target RNAs. RBPs contain RNA binding domains and bind to RNA molecules via specific sequence motifs. RNP complexes have various functions in gene expression including messenger RNA (mRNA) trafficking, RNA processing and silencing. In neurons, RBPs deliver specific sets of mRNAs to subcellular compartments such as axons and dendrites to be locally translated. Mutations or deletions in genes coding for RNPs have been reported as causes for neurological disorders such as SMA, ALS, and FXS. As RBPs determine axonal or dendritic mRNA repertoires as well as proteomes by trafficking selective mRNAs and regulating local protein synthesis, they play a crucial role for neuronal function. In this review, we summarize the role of well-known RBPs, SMN, TDP-43, FUS, and FMRP, and review their function for local protein synthesis in neurons. Furthermore, we discuss their pathological contribution to the neurological disorders.

## Introduction

Dysfunctional RNA processing in neuronal tissue is often observed in neurodegenerative diseases and plays a crucial role in neuronal pathology. This implies the essential part of ribonucleoprotein (RNP) complexes, that play an important role in RNA metabolism. RNP complexes contain RNA molecules with specific sequences/motifs bound to RNA-binding proteins (RBP). RNPs regulate eukaryotic gene expression in various cellular mechanisms including formation of ribosomes, spliceosomes and RNA-induced silencing complexes (RISCs) (Dreyfuss et al., 2002). In RNP complexes, RNAs and RBPs influence the fate of each other. On the one hand, RBPs act on processing, modification, stability, translation, and localization of RNAs, on the other hand, RNAs can regulate function, interaction, stability, and localization of RBPs. One example are long non-coding RNAs (lncRNAs). They can regulate the function of RBPs by recruiting transcription factors or chromatin-modifying complexes to chromatin (Cech and Steitz, 2014). One of the most well-characterized RNPs are messenger RNPs (mRNPs). They are composed of specific mRNAs and their mRNA-binding proteins (mRBPs). Assembly of the mRNPs can influence subcellular localization, post-transcriptional modification and translation of the transcripts. The misregulated assembly of mRNPs in the central nervous system (CNS) often leads to neurodegenerative diseases such as amyotrophic lateral sclerosis (ALS) and spinal muscular atrophy (SMA) (Shukla and Parker, 2016). In pathology, alterations in RNA metabolism manifest itself in misregulated splicing, adenylation, transcription, mRNA transport, translation, and modified decay. Those changes can be caused by various defects including reduced RBP expression, aggregation-prone RBPs or RBP sequestration in RNA foci. A detailed summary about general mechanisms involved in RNA metabolism in pathology can be found in a review by Nussbacher et al. (2019).

Due to the extreme cellular compartmentalization of neurons, it is essential to provide local mRNA transcripts and this especially makes neurons vulnerable to loss of RNPs. It has been suggested that neurons can regulate protein homeostasis at subcellular levels. Numerous evidences support this hypothesis that neurons can synthesize proteins at the synaptic compartment in response to extra stimuli. For example, the cellular components necessary to produce proteins are detected at the synaptic area such as ribosomes and mRNAs (Ainsley et al., 2014; Scarnati et al., 2018; Poulopoulos et al., 2019). In this scheme, RNPs deliver specific sets of mRNAs and produce repertoires of mRNAs in subcellular compartments, resulting in unique synaptic proteomes in neurons. Therefore, accurate function of RNPs and efficient local mRNA translation are crucial for neuronal development and function.

This review focuses on translational abnormalities caused by defects in mRNPs in neurological disorders including SMA, ALS, frontotemporal dementia (FTD), and fragile X syndrome (FXS). Table 1 summarizes the reviewed RBPs, the resulting neurodegenerative disorder as well as the underlying molecular mechanisms leading to pathology.

**Table 1:** Summary of RNPs in neurodegenerative disorders.

**Disease**	**RBP**	**Gene**	**Mechanism**
Spinal muscular atrophy (SMA)	Survival motor neuron (SMN)	Homozygous deletions, loss-of function mutations	Reduced RBP expression
Amyotrophic lateral sclerosis (ALS), Frontotemporal dementia (FTD)	TAR DNA binding protein 43 (TDP-43), Fused in sarcoma/translocated in liposarcoma (FUS/TLS)	Mutations, mislocalization (abberant methylation)	Aggregation- prone RBP, RBP sequestration in RNA foci
Fragile X syndrome (FXS)	Fragile X mental retardation protein (FMRP)	CGG>_200_ repeat expansion in the 5′ UTR of *FMR1*	Reduced RBP expression, RBP sequestration in RNA foci

## Protein Synthesis in Neurons

Protein synthesis is an essential cellular process for function, development and survival. In this process, a coordinated interplay between proteins and RNA is crucial to produce functional proteins. Mutations in genes involved in RNA metabolism lead to various diseases. Interestingly, many of these mutations are associated with neurodegenerative, neurodevelopmental and neuromuscular disorders. Because neurons are post-mitotic, highly polarized and larger in size compared to other types of cells, less severe mutations in genes involved in RNA metabolism may cause dysfunction in neurons, but other types of cells might tolerate these mutations. Dysfunction of RNA-related processes including tRNA synthesis, ribosome biogenesis, sequestration and mislocalization of mRNAs has been reported as pathological mechanisms in neurological disorders (Jordanova et al., 2006; Butterfield et al., 2014; Rossi et al., 2015). Furthermore, mutations in genes involved in RNA metabolisms are also reported as causative mutations in neurodegenerative diseases. For example, deletion of a gene coding for fragile X mental retardation protein (FMRP, a repressor of mRNA translation) causes mental retardation and mutations in a gene coding for TAR DNA binding protein (TDP-43), which impairs protein synthesis by sequestering mRNAs, can cause ALS or frontotemporal dementia (Verkerk et al., 1991; Coyne et al., 2017).

Ribosomes are crucial components of mRNA translation. Recent data suggests that ribosomes are rather heterogeneous and this may affect protein synthesis efficiency in subcellular compartments (Simsek et al., 2017; Genuth and Barna, 2018). Mutations in genes important for ribosome biogenesis such as ribosomal protein and ribosomal RNA can cause neurodegenerative disorders (ribosomopathies) (Farley-Barnes et al., 2019). Besides the conventional cap-dependent translation initiation mechanism, Internal Ribosome Entry Site (IRES)- mediated translation is reported in neurons (Pinkstaff et al., 2001; Pelletier and Sonenberg, 2019). While the pathogenic mechanism is unclear, it has been shown that TAU, which contributes to Alzheimer's pathology, is synthesized via IRES mediated translation initiation (Veo and Krushel, 2009). In addition, another non-conventional translation mechanism has been reported, repeat-associated non-AUG (RAN) translation. For example, a pathological expansion of trinucleotide repeats in *C9ORF72* forms IRES like structures in RNA, producing toxic dipeptide repeats, which form aggregates in neurons (Ash et al., 2013; Zu et al., 2013). These dipeptide-containing aggregates are observed in ALS patient samples with mutations in *C9ORF72* (Zu et al., 2013). Taken together, these findings highly suggest that protein synthesis is a particularly important process in neurons and its dysregulation can cause neuronal dysfunction.

## Local mRNA Translation in Axons and Dendrites

In neurons, mRNAs can be transported to axons and dendrites as mRNPs and locally translated to maintain local protein homeostasis. The localization sequence (also called zip code) to neuronal projections is often located in the 3′-untranslated region (UTR) of the mRNA (Andreassi and Riccio, 2009). It is believed that activity-dependent synaptic protein synthesis is crucial for learning and memory formation (Buffington et al., 2014). Cellular components necessary for regulating protein synthesis have been found in the neurite compartments including mRNAs, tRNAs, ribosomes, translation factors, the RNA-induced silencing complex (RISC) as well as microRNAs (miRNAs) (Tiedge and Brosius, 1996; Lugli et al., 2005; Kye et al., 2007; Ainsley et al., 2014; Scarnati et al., 2018; Poulopoulos et al., 2019). Local mRNA repertoire has been profiled in many different contexts by various methods (Matsumoto et al., 2007; Poulopoulos et al., 2019). In brief, transcriptomes and proteomes in growth cones isolated from cerebral cortices have been profiled by RNA sequencing and mass spectrometry (Poulopoulos et al., 2019). This data suggests that subcellular transcriptomes/proteomes play an important role in neural circuit formation and more interestingly, subcellular transcriptome distribution is mTOR dependent. In addition, dendritic RNAs have been identified from the synaptosomes by microarray analysis and their subcellular distribution can be altered by neuronal activity (Matsumoto et al., 2007). These data sets confirm that neuronal projections, either axons or dendrites contain different sets of mRNAs compared to soma. These findings highly suggest that local proteomes are supplied by local mRNAs. Recently, it has been identified that mitochondria and late endosomes play an important role for protein synthesis at the synaptic site (Cioni et al., 2019; Rangaraju et al., 2019). Due to these reports, late endosomes serve as a platform of axonal local translation by binding to RBPs, ribosomes and mRNAs. Furthermore, the proteins important for mitochondrial integrity are produced on the late endosomes (Cioni et al., 2019). Additionally, while it has been suggested that synaptic energy can be supplied by glycolysis in presynaptic boutons (Ashrafi et al., 2017), mitochondria seem to be the sole energy source for post-synaptic plasticity as mitochondria-depleted dendritic spines show clearly reduced levels of plasticity induced protein synthesis as well as spine growth (Rangaraju et al., 2019).

Another interesting cellular component regulating neuronal protein synthesis are RNA granules. Neurons contain various RNA granules including stress granule, processing body (P-body) and tRNP granule (Anderson and Kedersha, 2009). It has been suggested that RNA granules can repress protein synthesis and store mRNAs until they have reached their final subcellular destination in neurons (Kiebler and Bassell, 2006). Importantly, it has been shown that dendritically located RNP granules can be disassembled in an activity-dependent manner and mRNAs can be used as a template to produce synaptic proteins (Krichevsky and Kosik, 2001). This process is crucial for neuronal health and function as it is a cellular homeostatic mechanisms to handle external stress and control synaptic plasticity. While many RBPs are involved in assembly and disassembly of RNA granules, here we will focus on the RBPs whose dysfunction is associated with neurological disorders.

## Axonal RNA Binding Proteins and Axonal Local Translation

It is still not fully understood how (or even whether) proteins can be synthesized in mature axons *in vivo*, and whether this is a universal mechanism happening in all kinds of neurons or if it is a neuronal subtype specific phenomenon. However, increasing numbers of evidence suggest that active protein synthesis happens in growing and regenerating axons (Spaulding and Burgess, 2017). To characterize the axonal local translation mechanism, different approaches have been made including profiling axonal transcriptome/proteome and identifying RNA binding proteins for mRNA trafficking or splicing (Briese et al., 2018; Poulopoulos et al., 2019; Zhang et al., 2019). One of the most important player of protein synthesis, the ribosomes, are detected in axonal compartments (Akins et al., 2017). Axonal ribosomes can be trafficked from soma, but they can also be transferred from surrounding cells such as glia or Schwann cells via exosomes (Court et al., 2008; Muller et al., 2018). Another important component of translation are messenger RNAs (mRNAs). As mentioned above, RBPs bind to mRNAs selectively, localize them in subcellular compartments and even regulate protein synthesis. This will impact on local proteome composition. For example, the zip code binding protein 1 (ZBP1) binds the 3′-UTR of β-actin mRNA and hinders protein synthesis by blocking translation initiation as long as β-actin mRNA is not localized to its destination (Huttelmaier et al., 2005). Next, mTOR kinase is an essential regulator of mRNA translation in injured axons (Terenzio et al., 2018). The mTOR kinase can regulate the efficiency of protein synthesis upon extracellular signals or intracellular status via turning on signaling cascades. Finally, numerous miRNAs and protein components of RISC have been detected in axons and growth cones (Hengst et al., 2006; Kye et al., 2014; Gershoni-Emek et al., 2018). This data implies that miRNAs can function as a translational repressor in axons. For example, it has been shown that miR-183 can repress protein synthesis of mTOR by direct binding to 3′UTR of *mTOR* mRNA in axons (Kye et al., 2014). Taken together, with current knowledge, we can conclude that proteins can be synthesized in axonal compartments.

### Spinal Muscular Atrophy (SMA)

**Figure 1:**
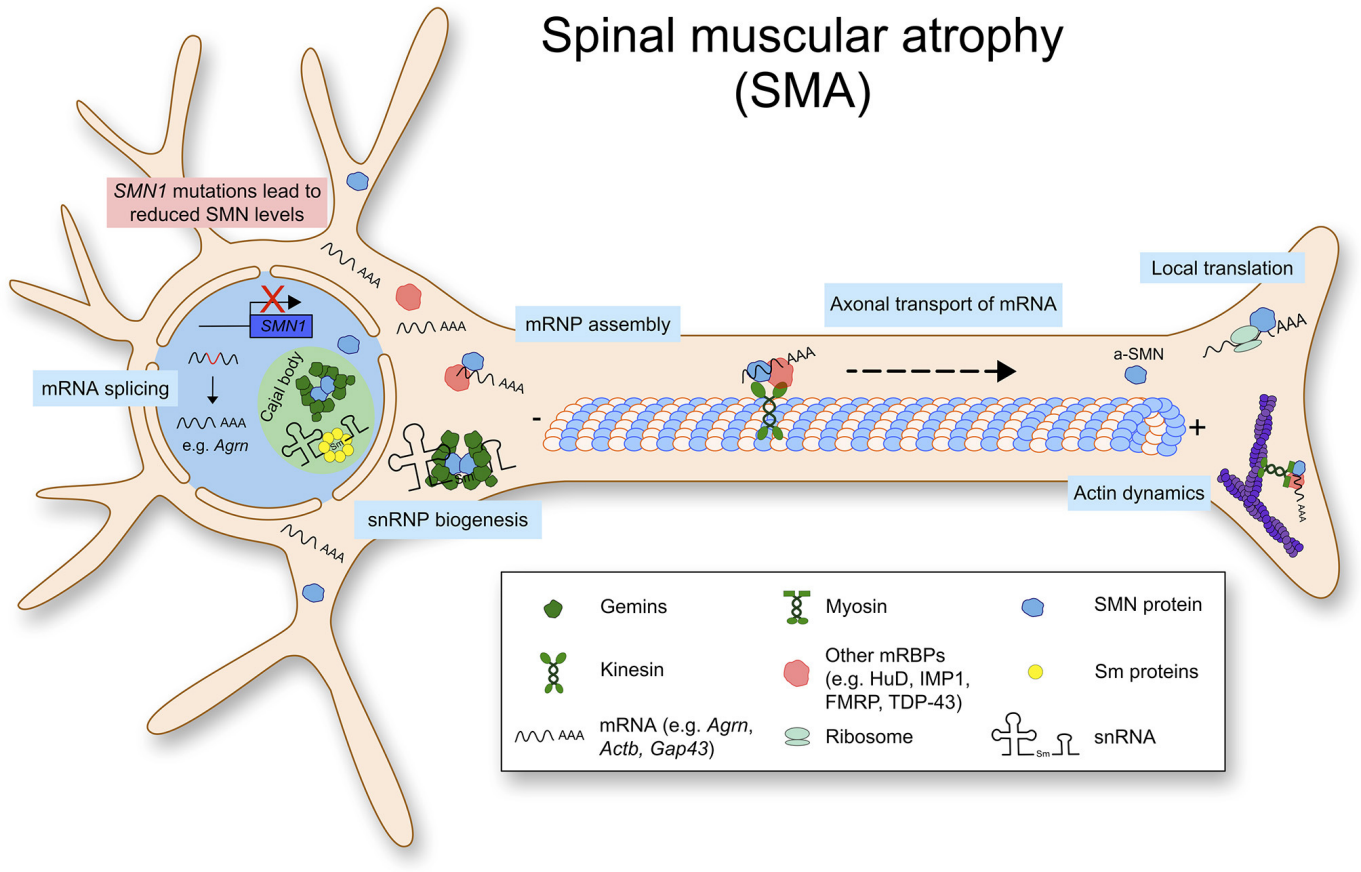
Spinal muscular atrophy (SMA) is caused by mutations or deletions in the *SMN1* gene, that lead to reduced SMN protein levels. Physiologically, SMN is mostly found in a complex. Besides snRNP biogenesis and splicing, SMN has important functions such as mRNP granule assembly, axonal mRNA transport and local translation.

SMA is caused by a reduction of the ubiquitously expressed SMN protein which leads to loss of lower motor neurons. In more than 95% of SMA cases, homozygous deletions are found in the *SMN1* gene encoding the full length SMN protein. Humans possess a duplication of the *SMN1* gene, *SMN2*, which produces a transcript lacking exon 7 due to a single point mutation located in exon 7 (Lorson et al., 1999). Due to this, *SMN2* encodes for a unstable SMN protein, which is rapidly degraded by the proteasome pathway (Chang et al., 2004). Therefore, the copy number of *SMN2* can determine the amount of SMN proteins and the severities of SMA (Butchbach, 2016). Importantly, complete loss of SMN is embryonically lethal in multiple organisms including humans and mice (Schrank et al., 1997). Therefore, SMA mouse models are generated by introducing human *SMN2* genes in murine *Smn*-null background. Two copies of *SMN2* in *Smn* null background causes severe phenotypes in mice mimicking type I SMA patients. In this model, mice are born with a normal number of motor neurons, but lose 3–40% of spinal cord and lower brainstem motor neurons by post-natal day 5 (Monani et al., 2000). It is still under investigation how a ubiquitously expressed protein like SMN leads to specific loss of lower motor neurons in SMA. It has been hypothesized that specific sets of primary mRNAs are dysregulated in SMA and that they are crucial for development and survival of motor neurons. For example, Agrin (*Agrn*) is mis-spliced in motor neurons of SMA mice, and notably it is important for organizing acetylcholine receptors at the NMJ (Zhang et al., 2013; Kim et al., 2017). Furthermore, an alternatively spliced form of SMN, the axonal-SMN (a-SMN) has been reported. This isoform seems highly expressed in the axonal compartment of motor neurons and it enhances axon growth in neurons. While the molecular mechanism underlying the role of a-SMN in axonogenesis remains still unclear, this might hint at the selective phenotype of SMN loss in motor neurons (Setola et al., 2007). Collectively, the functions of SMN with respect to RNA homeostasis are shown in Figure 1.

Splicing correction of *SMN2* using an antisense oligonucleotide (ASO) has been developed to restore SMN protein levels. Briefly, ASO binds to an intronic silencer in intron 7 and thereby it enhances the inclusion of exon 7 of *SMN2* (Hua et al., 2008, 2011). Noteworthy, this ASO-mediated treatment, named Nusinersen became the first Food and Drug Administration (FDA) approved treatment for SMA. In addition, gene therapy using AAV9 has been also FDA-approved as a SMA therapy. As SMA is caused by deficiency of SMN protein, SMN protein can be restored in motor neurons by viral delivery (Mendell et al., 2017).

#### Survival Motor Neuron (SMN) for Local mRNA Translation

As mentioned above, SMA is caused by SMN protein deficiency. SMN is an RNA binding protein, ubiquitously expressed and performs multiple essential cellular functions, especially in RNA processing. SMN protein levels are higher during embryogenesis and decrease after birth (Jablonka and Sendtner, 2017). Correlating with SMN levels, assembly of small nucleolar ribonucleoproteins (snRNP) declines in mouse spinal cord during the early postnatal weeks (Gabanella et al., 2005). Basic SMN complex consists of SMN and Gemin2-8, and SMN protein itself is rather unstable without its binding partners (Lorson et al., 1998; Otter et al., 2007). Reactive oxygen species (ROS) can inhibit SMN complex formation. Even subtoxic levels of ROS can inhibit SMN complex formation by inducing intramolecular disulfide bridging (Wan et al., 2008). Activity of the SMN complex is regulated by phosphorylation, and phosphorylation of SMN enhances cytoplasmic localization and snRNP biogenesis (Grimmler et al., 2005; Renvoise et al., 2012). The SMN complex can be detected in the cytoplasm, nucleus and nuclear gems (Gemini of Cajal bodies) (Carvalho et al., 1999). SMN levels are especially higher in nuclear gems, which consist of RNAs and proteins. It has been suggested that the composition of SMN complex can be different in neurite compartments of neurons (Todd et al., 2010).

SMN complex is a molecular chaperone, regulating structure, biogenesis and function of snRNPs that are involved in splicing (Liu and Dreyfuss, 1996). For snRNP biogenesis, SMN facilitates the assembly of Sm proteins onto the Sm site of the snRNA and produces functional snRNPs (also known as spliceosomes). SMN also functions in the transport and assembly of other ribonucleoprotein classes, including microRNPs or telomerase RNPs (Buhler et al., 1999; Mourelatos et al., 2001, 2002).

SMN is involved in mRNA processing after being assembled into mRNPs. These additional functions range from nuclear export, intracellular trafficking to translation. In neurons, SMN binds to mRNAs directly or via other mRBPs, and transports polyA-tailed mRNAs to the axonal compartment to be locally translated (Akten et al., 2011; Fallini et al., 2011; Ottesen et al., 2018). Such mRBPs include HuD (ELAVL4, ELAV like RNA binding protein 4), KSRP (KH-type splicing regulatory protein), hnRNPs (heterogeneous nuclear ribonucleoproteins) and IMP1 (also known as IGF2BP1, Insulin like growth factor 2 mRNA binding protein 1) (Tadesse et al., 2008; Akten et al., 2011; Dombert et al., 2014; Fallini et al., 2014). The known *Smn* mRNA cargo in neurons include mRNAs of β-actin, growth-associated protein 43 (*Gap43*), annexin 2 (*Anxa2*), and neuritin*/cpg15* (Akten et al., 2011; Fallini et al., 2016; Rihan et al., 2017; Ottesen et al., 2018). Stable supply of β-actin protein seems important for growth of neurons as a building block, GAP43 is crucial for axonal pathfinding and regeneration, CPG15 is a known neurotrophic factor, and ANXA2 is also important for growth of cells. It is also worth to note that rapid bidirectional transport of SMN protein has been observed in primary neurons and it is microtubule dependent (Zhang et al., 2003). Taken together, these data collectively suggest that SMN plays an important role for local proteome composition by trafficking mRNAs, to be translated in the axonal compartment.

### Amyotrophic Lateral Sclerosis (ALS) and Frontotemporal Dementia (FTD)

ALS is an adult-onset, progressive neurodegenerative disorder causing deterioration and death of upper and lower motor neurons (Cleveland and Rothstein, 2001). Most of ALS patients die from respiratory failure within 3–5 years after onset (Swinnen and Robberecht, 2014). The majority of ALS cases (90–95%) is considered as sporadic ALS (sALS) without family history and 5–10% of cases show Mendelian inheritance. Over the last decades, genetic variants in over 25 genes are associated with ALS (Nguyen et al., 2018). The most common and most well established mutations contributing to ALS pathology are found in Cu-Zn superoxide dismutase 1 (*SOD1*), *TDP-43, FUS* and a hexanucleotide expansion repeat in Chromosome 9 Open Reading Frame 72 (*C9orf72*) (Chia et al., 2018). Mutations in *SOD1* are the first reported ALS- associated mutations and are the second most common mutations in ALS (Rosen et al., 1993). Those mutations in *SOD1* can be found in about 12% of familial ALS (fALS) and around 1% sALS (Renton et al., 2014). The most common mutated gene in ALS and FTD is *C9orf72*, which forms highly stable RNA G-quadruplexes due to a hexanucleotide repeat in the 5′ non-coding region (Renton et al., 2011). Up to now, the protein function of C9ORF72 is rather unknown, but the formation of G-quadruplexes are involved in sequestering RNA metabolism such as telomere stability, transcription, splicing, RNA transport, and translation in RNA foci (Fratta et al., 2012). More importantly, a hexanucleotide repeat in the 5′ non-coding region of *C9orf72* can produce dipeptide repeat proteins (Gly-Ala) via RAN translation. These dipeptide proteins form aggregates in neurons and cause cellular toxicity (May et al., 2014). In 2008, mutations in the gene coding for the transactive response DNA-binding protein 43 (TDP-43) have been reported as disease causing mutations in ALS (Sreedharan et al., 2008). A recent study showed that mutations in TDP-43 account for 4% of fALS and for <1% of sALS cases (Chia et al., 2018).

**Figure 2:**
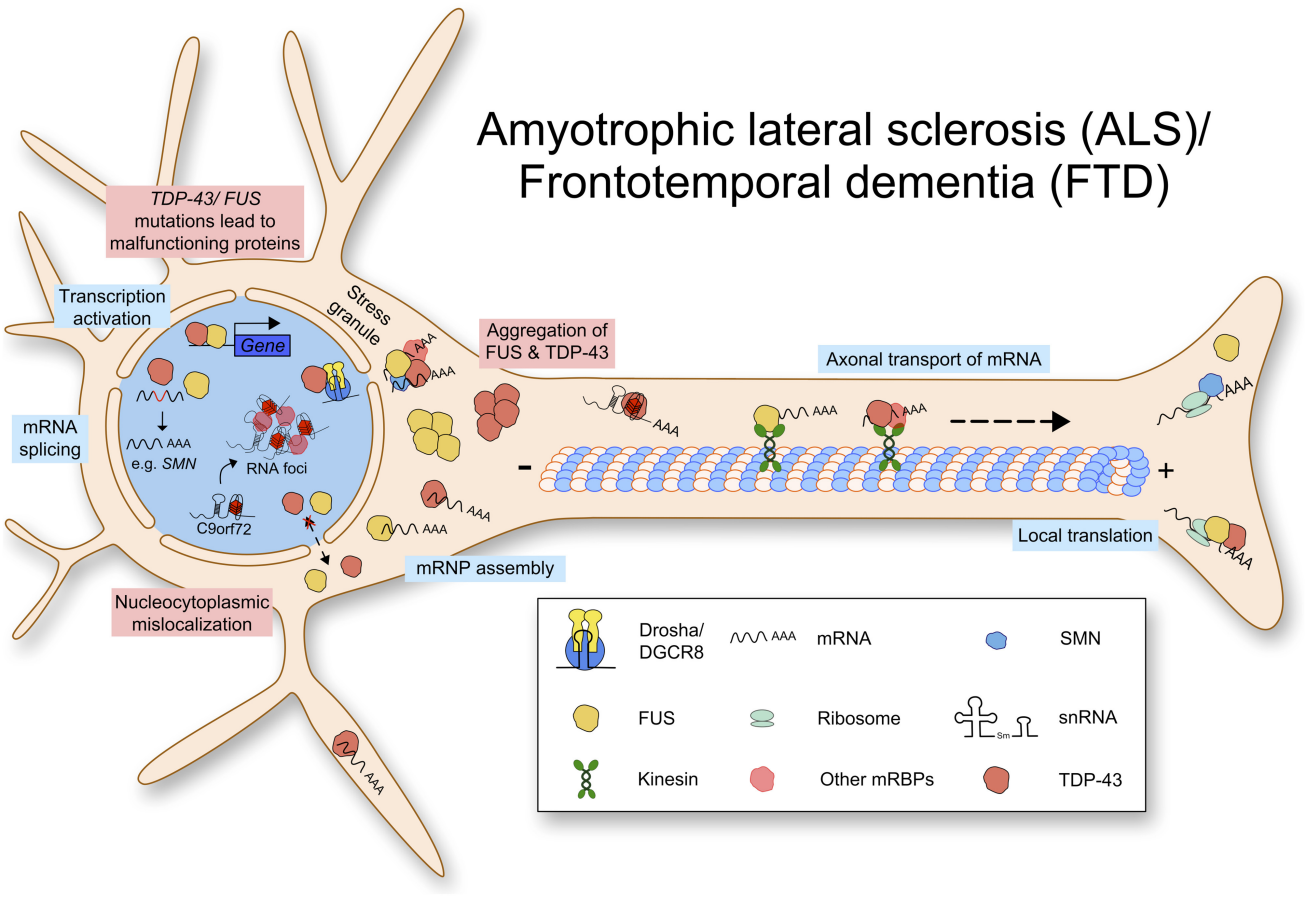
Amyotrophic lateral sclerosis (ALS) and Frontotemporal dementia (FTD) are caused by multiple mutations leading to defects in RNA metabolism. Mutated TDP-43 or FUS protein aggregates in the cytoplasm and lacks nuclear import. TDP-43 and FUS function physiologically in transcription activation, mRNA splicing, mRNP assembly, axonal transport of mRNA, and local translation. Due to loss of normal protein function and gain of toxic function, RNA metabolism is impaired.

In addition to ALS, mutations in TDP-43 can lead to frontotemporal lobar degeneration (FTLD-TDP), which is characterized by insoluble inclusions containing TDP-43 aggregates (Seelaar et al., 2007). TDP-43 proteinopathies show various molecular phenotypes including loss of nuclear expression and pathological aggregations (Winton et al., 2008; Zhang et al., 2009). The molecular mechanisms contributing to ALS and FTD pathology are summarized in Figure 2. At the moment, it is unclear whether TDP-43 mediated neurodegeneration is caused by the loss of function or the gain of toxic function.

#### Transactive Response DNA-Binding Protein 43 (TDP-43) for Local mRNA Translation

TDP-43 is a 43 kDa highly conserved, ubiquitously expressed DNA/RNA-binding protein, encoded by the *TARDP* gene in humans. It plays an important role in RNA processing and its mutations are linked to neurodegeneration (Lee et al., 2011). TDP-43 binds to a large variety of transcripts including mRNAs and non-coding RNAs (Sephton et al., 2011; Tollervey et al., 2011). Interestingly, some of those transcripts are related to neuronal development or synaptic activity (Polymenidou et al., 2011). Furthermore, TDP-43 also regulates microRNA (miRNA) biogenesis by binding to the Drosha-DGCR8 (DiGeorge syndrome critical region gene 8) microprocessor complex, which is important for processing primary miRNAs. Indeed, knockdown of TDP-43 results in altered levels of miRNAs (Kawahara and Mieda-Sato, 2012).

**Figure 3:**
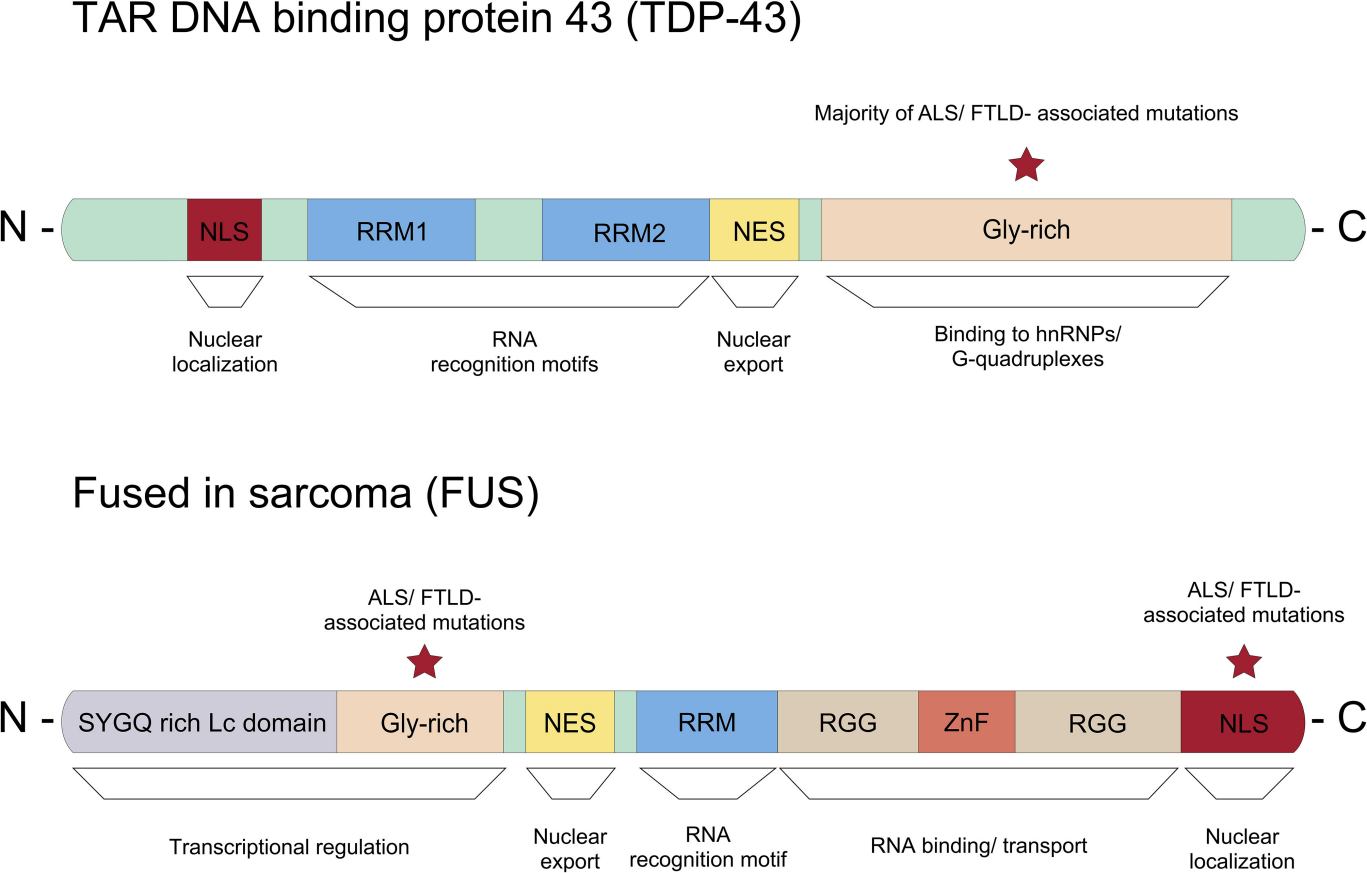
Representation of the protein domain structure of TDP-43 and FUS. The majority of ALS/FTD associated dominant missense mutations in TDP-43 can be found in the glycine-rich c-terminus. Whereas, most ASL/FTLD associated mutations in FUS are described in the nuclear localization signal (NLS).

In addition to the RNA binding motifs, TDP-43 contains a C-terminal low complexity domain, where the majority of ALS-associated dominant missense mutations are located (Figure 3) (Pesiridis et al., 2009). It has been shown that TDP-43 binds to the hnRNPs including hnRNP A1, hnRNP A2/B1, hnRNP A3, and hnRNP C1/C2 via this glycine-rich domain (Buratti et al., 2005; Freibaum et al., 2010). Interestingly, TDP-43 regulates splicing of several transcripts including *Smn* mRNA (Bose et al., 2008). Various stressors mediate the redistribution of TDP-43 from the nucleus into the cytoplasm, where it is associated with RNAs and recruited to stress granules (Colombrita et al., 2009; Dewey et al., 2011; Mcdonald et al., 2011).

In neurons, TDP-43 binds to specific RNA structures, G-quadruplex in mRNAs and deliver them to neurites to be translated locally. Interestingly, TDP-43 protein with an ALS-linked mutation in the C-terminal glycine-rich domain diminishes the binding affinity to G-quadruplex in mRNAs and their translation in neurites (Ishiguro et al., 2016). Furthermore, ALS associated mutations in TDP-43 impairs mRNA trafficking in axons (Alami et al., 2014). Taken together, these data imply that disturbed local mRNA translation contributes to pathology of ALS. It is worthy to note that TDP-43 is also crucial for regulating dendritic local translation in response to neuronal activity (Endo et al., 2018). This may suggest the role of TDP-43 for psychiatric outcomes observed in FTD patients.

#### Fused in Sarcoma/Translocated in Liposarcoma (FUS) for Local mRNA Translation

Fused in sarcoma/Translocated in liposarcoma (FUS/TLS) is a DNA/RNA-binding protein and mutations in FUS are implicated in ALS (about 4% of fALS and <1% of sALS) and FTLD (Chia et al., 2018). FUS can shuttle between the nucleus and cytoplasm, and is detected at the pre-synaptic site of hippocampal neurons and at the neuromuscular junction (NMJ) (Schoen et al., 2015; So et al., 2018). FUS plays various cellular functions including transcriptional activation, RNA splicing, mRNA trafficking and translation, and DNA repair as well as genome stability by direct binding to nucleic acid molecules (Efimova et al., 2017). It has been shown that FUS can act on mRNA trafficking and local translation in neurons. Importantly, ALS-causing mutant FUS harboring neurons showed impaired axonal protein synthesis (Lopez-Erauskin et al., 2018). Additionally, FUS protein has been detected in stress granules, which is known to function in translation repression (Bosco et al., 2010). However, it is still unclear whether wild-type FUS can be assembled into stress granules or this is an exclusive phenotype of mutant FUS (Aulas and Vande Velde, 2015).

Several mutations in FUS have been associated with ALS/FTD. Unlike TDP-43, where the majority of mutations linked to ALS/FTD are located in the low complexity domain, most ALS/FTLD-associated mutations are found in the C-terminal nuclear localization signal (NLS) in FUS (Figure 3) (Da Cruz and Cleveland, 2011). Due to mutations in the NLS, mutant FUS proteins seem mis-localized and form inclusions in the cytoplasm (Vance et al., 2009). Proteomic approaches showed that these inclusions contain various proteins important for mRNA splicing, RNA processing and transport such as eIF4A3 (eukaryotic translation initiation factor 4A3) and eIF3 (eukatyotic translation initiation factor 3). Importantly, FUS regulates mRNA translation and non-sense mediated decay (NMD), and mutations in FUS suppress protein synthesis efficiency and cause hyperactive NMD resulting in impaired protein homeostasis in cells (Kamelgarn et al., 2018). In summary, mutant FUS induces inclusions, and this sequesters important proteins for cellular processes such as mRNA translation and NMD, which can be detrimental to cellular function and survival.

Interestingly, it has been shown that motor and cognitive deficits in ALS are caused by increase of mutant FUS in axonal compartments, that lead to reduced intra-axonal translation and therefore to synaptic dysfunction (Lopez-Erauskin et al., 2018). This decreased protein synthesis is not accompanied with FUS aggregation or loss of nuclear FUS function, suggesting a new patho-mechanism caused by mutant FUS.

Notably, FUS can interact with TDP-43. This interaction is increased by ALS-associated mutations in TDP-43 (Ling et al., 2010). Therefore, mutations in TDP-43 and FUS might share a common mechanism leading to ALS. In addition, FUS can interact with SMN and their binding affinity to each other increases by mutations in FUS. Furthermore, FUS inclusions sequester cytoplasmic SMN, resulting in reduced axonal SMN levels (Groen et al., 2013). As SMN delivers mRNAs to axons and growth cones for local translation (Akten et al., 2011), this data suggests that mutations in FUS induce impaired axonal local translation by sequestering the axonal SMN-mRNA complex. Taken together, these data imply that ALS and SMA might have a common patho-mechanisms such as decreased mRNP activity by SMN and impaired axonal local translation. Similar to TDP43, the gain of toxic function and/or loss of normal function in FUS contributes to ALS pathology.

## Dendritic RNA Binding Proteins and Local mRNA Translation

### Fragile X Syndrome (FXS)

Fragile X syndrome (FXS) is a genetic disorder with intellectual disability caused by loss/deficiency of fragile X mental retardation protein, FMRP. FMRP is a polyribosome-associated RNA binding protein, which is encoded by the fragile X mental retardation 1 gene (*FMR1*) (Flannery et al., 1995). Mutations in the *FMR1* gene are also associated with other diseases including the fragile x-associated tremor/ataxia syndrome, premature ovarian aging and the polycystic ovarian syndrome (Kenneson and Warren, 2001). Mostly, loss of FMRP protein is caused by a trinucleotide repeat expansion in the 5′-UTR of *FMR1*. When this triple repeat exceeds 200 repeats, it is methylated together with the *FMR1* promoter, resulting in transcriptional repression. In addition, missense mutations leading to FMRP dysfunction have been identified as a cause for physical disability as well as cognitive and behavioral impairments (Sitzmann et al., 2018; Maddirevula et al., 2019).

#### Fragile X Mental Retardation Protein (FMRP) for Local mRNA Translation

FMRP contains three RNA binding domains; two ribonucleoprotein K homology domains (KH domains) and one RGG domain, which can bind to poly-riboguanylic acid (Ashley et al., 1993). FMRP is a well-described translational repressor in neurons and FMRP deficient neurons show a delayed maturation and abnormal morphology of the dendritic spines (Comery et al., 1997; Weiler and Greenough, 1999). While there are exceptions, FRMP represses mRNA translation via direct binding to G-quadruplexes in target mRNAs (Schaeffer et al., 2001). Interestingly, it has been shown that translation can be inhibited by FMRP binding to the inter-subunit space of the ribosome and this process is independent of G-quadruplexes on the target mRNA (Chen et al., 2014).

While we discuss the role of FMRP in dendritic local translation in this review, FMRP can be detected in axons together with microRNAs and mRNAs, suggesting its role for axonal local translation (Hengst et al., 2006). In sensory neurons, FMRP granules delivers mRNAs such as *Map1b* and *Calm1* to axonal compartments, and release them to be translated upon stimulation by nerve growth factor (NGF). This process is important to NGF induced axonal outgrowth (Wang et al., 2015).

**Figure 4:**
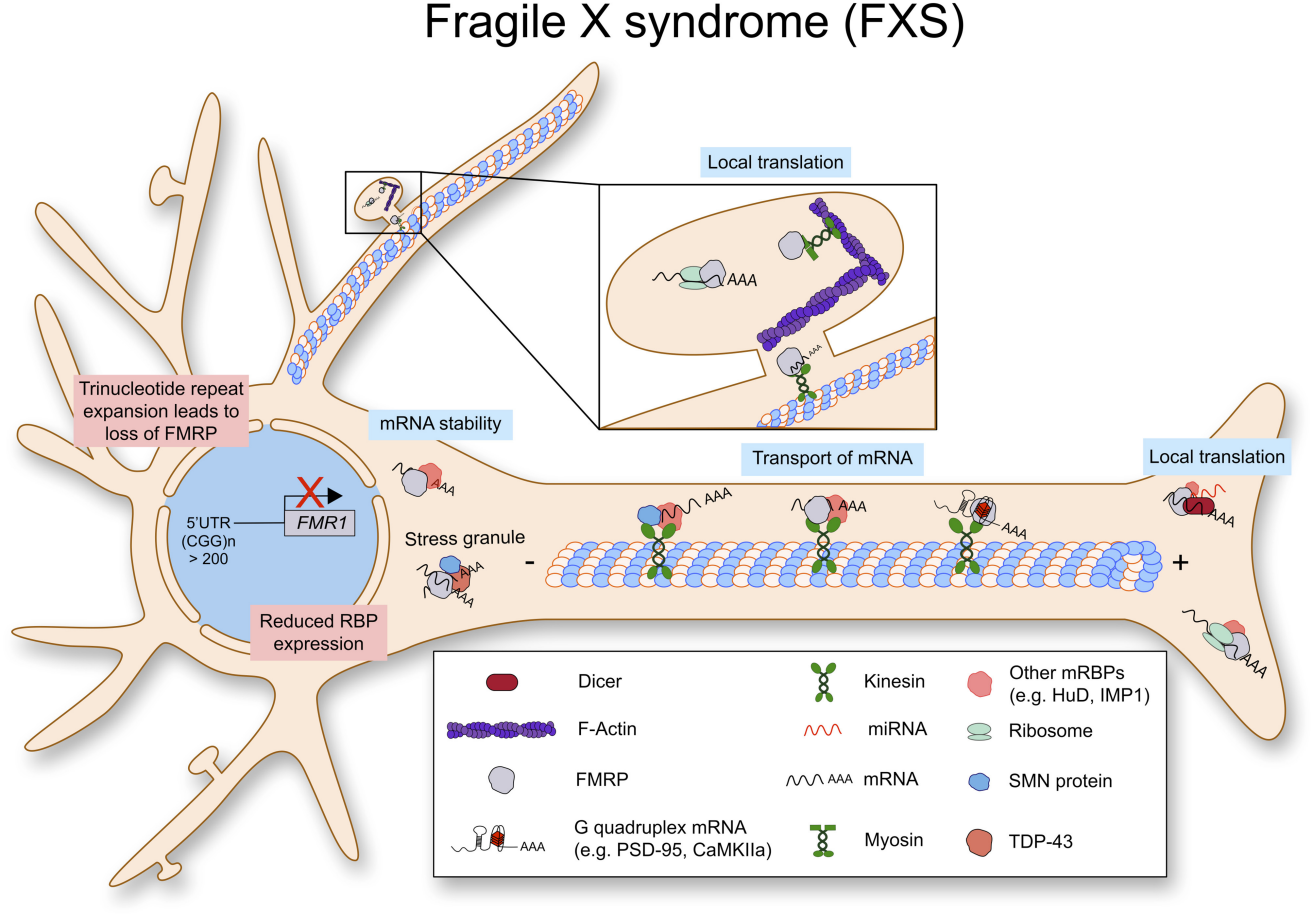
Fragile X syndrome (FXS) is caused by a trinucleotide repeat expansion in the 5′-UTR of the *FMR1* gene. As a result, the RNA binding protein FMRP is lost. FMRP exhibits multiple functions in RNA metabolism as it stabilizes mRNA, transports them along the axon to be locally translated.

The cellular mechanism of dendritic local translation has been intensively studied as well as the role of FMRP in postsynaptic protein synthesis. FMRP participates in dendritic local translation in various steps (Figure 4). First, as mentioned above, FMRP binds to certain sets of mRNAs and delivers them to dendritic compartments. The experimentally validated FMRP binding mRNAs include its own mRNA *Fmr1, Dlg4* (PSD-95), and *Mmp-9* (Matrix metalloprotease 9) (Schaeffer et al., 2001; Zalfa et al., 2007; Janusz et al., 2013; Ifrim et al., 2015). Surprisingly, high throughput sequencing of RNAs isolated by crosslinking immunoprecipitation (CLIP) assay has revealed that FMRP binds to 842 mRNAs, and 32% of them code for synaptic proteins (Darnell et al., 2011). This work has been further confirmed by independent experiments (Ascano et al., 2012; Maurin et al., 2018). Second, FMRP can repress translation by binding to mRNAs via non-coding RNA BC1. In this case, FMRP doesn't bind to mRNA directly, but BC1 mediates FMRP function on translation (Zalfa et al., 2003). Third, FMRP can stall ribosomes on mRNAs. It seems that FMRP binds to ribosomes and inhibits the elongation process (Darnell et al., 2011; Chen et al., 2014). Fourth, FMRP binds to RNA induced silencing complex (RISC) and regulates microRNA function for translational repression (Muddashetty et al., 2011). In brief, FMRP regulates mRNA translation of PSD-95 via miR-125a containing RISC. Finally, post-translational modifications of FMRP also play an important role in mRNA translation via mRNA granule assembly. Recent papers suggest that phosphorylation of FMRP induces mRNA granule formation, which can sequester mRNAs and proteins, resulting in inhibition of new protein synthesis. Instead, methylation of FMRP in its arginine-glycine rich (RGG) domain can cause disassembly of mRNA granules leading to translational activation (Tsang et al., 2019). While further *in vivo* studies are required, this data unveiled a new cellular mechanism underlying neuronal activity induced FMRP-mediated protein synthesis at the synaptic site. Taken together, FMRP regulates local mRNA translation in neurons via multiple layers of cellular mechanisms.

## Discussion and Summary

Here, we have reviewed the current knowledge about the role of RNA binding proteins in local protein synthesis in subcellular compartments of neurons. While there are many more RNA binding proteins in neurons and their roles are equally important and interesting, we have only covered the most well-known four proteins, SMN, TDP-43, FUS, and FMRP. Importantly, dysregulation of these proteins caused by mutations and deletions is often associated with human neurological disorders. As neurons are highly polarized, often large in size, and their function is transient, neuronal protein homeostasis needs to be regulated timely and spacially. Neurons solve this issue by quick production of proteins at local sites when new proteins are required. For this process, RNA binding proteins traffic mRNAs and store them in mRNP granules in synaptic compartments, and upon stimulus, mRNA can be released to produce new proteins. As this process is important for neuronal function, dysfunction of RNA binding proteins often cause neurological disorders, in the worst case, leading to organismal death. While many underyling cellular mechanisms have been characterized, the molecular functions and biophysical properties of RNA binding proteins are still not fully understood. Furthermore, it will be important to understand how dysfunctional RNA binding proteins and impaired local translation contribute to pathology of human diseases.

## Author Contributions

All authors listed have made a substantial, direct and intellectual contribution to the work, and approved it for publication.

### Conflict of Interest

The authors declare that the research was conducted in the absence of any commercial or financial relationships that could be construed as a potential conflict of interest.
